# Antibacterial Activity of MTA Fillapex and AH 26 Root Canal Sealers at Different Time Intervals

**DOI:** 10.7508/iej.2016.03.009

**Published:** 2016-05-01

**Authors:** Farnaz Jafari, Hossein Samadi Kafil, Sanaz Jafari, Mohammad Aghazadeh, Tahereh Momeni

**Affiliations:** a *Department of Endodontic, Dental School, Tabriz University of Medical Sciences, Tabriz, Iran; *; b * Drug Applied Research Center, Tabriz University of Medical Sciences, Tabriz, Iran; *; c * Department of Orthodontic, Dental School, Ilam University of Medical Sciences, Ilam, Iran;*; d *Department of Microbiology, Medical School, Tabriz University of Medical Sciences, Tabriz, Iran; *; e *Dentist, Dental School, Tabriz University of Medical Sciences, Tabriz, Iran*

**Keywords:** Antibacterial, *Enterococcus faecalis*, *Lactobacillus*, MTA Fillapex, *Sealers*, *Staphylococcus*

## Abstract

**Introduction:**

The main goal of endodontic treatment is elimination of bacteria and their by-products from infected root canals. This study compared the antibacterial effect of two different sealers, AH 26 and MTA Fillapex, on 4 microorganisms 24, 48 and 72 h and 7 days after mixing.

**Methods and Materials:**

The microorganisms used in this study consisted of *Lactobacillus acidophilus (*ATCC 4356), *Lactobacillus casei (*ATCC 39392)*, Staphylococcus aureus (*ATCC 25923) and* Enterococcus faecalis (*ATCC 29212). This test is based on the growth of bacteria and turbidity measurement technique using a spectrophotometer, and direct contact was conducted. Multiple comparisons were carried out using repeated-measures ANOVA followed by Tukey’s test and student’s t-test. The level of significance was set at 0.05.

**Results:**

The antibacterial activity in the indirect technique was more than the technique with both sealers. In the direct technique the antibacterial activity on all microorganisms were lower for MTA Fillapex sealer. In the indirect technique, both sealers exhibited similar antibacterial properties.

**Conclusion:**

The antibacterial effect of MTA Fillapex sealer was significantly less than that of AH 26 sealer in the direct technique. The antibacterial effects of both sealers were similar in the indirect technique.

## Introduction

Microorganisms and microbial products are the main etiologic factors involved in pulpal diseases and periapical lesions [1]. Therefore, the chief aim of endodontic treatment is to eliminate microbial agents from the infected root canals [2]. To achieve this aim, the root canals should be cleaned, shaped and obturated with sterilized materials with antimicrobial properties [3-5]. It is not always possible to completely eliminate microorganisms from the root canals [6] and microorganisms can also penetrate through coronal microleakage after obturation of the root canals [7]. 

*Enterococcus faecalis* (*E. faecalis*) is commonly isolated from primary [8, 9] and secondary endodontic infections [9-13]; however, its prevalence is higher in secondary endodontic infections [8]. *Staphylococcus *species are found in the structure of bacterial biofilm in endodontic infections and are resistant to the penetration of antibiotics [14]. *Lactobacilli *are gram-positive, anaerobic, asporogenous microaerophilic or facultative aerobics and are generally considered non-pathogenic [15, 16]. In infected root canals with chronic periodontitis the prevalence of *Lactobacilli* is 32% [17], while the prevalence of *Streptococci* is 40% [18]. 

Gutta-percha and various sealers, are used for obturation of the root canals. AH 26 (Dentsply, Tulsa Dental, Tulsa, OK, USA) with epoxy resin base is one of the most commonly used sealers in dentistry. This sealer has a high capacity to seal the root canals [19]. Release of formaldehyde by AH 26 sealer during its setting has been confirmed [20, 21]. These sealers do not have formaldehyde in their chemical composition but the chemical reactions between their constituents that occur during the setting phase result in the production and release of formaldehyde which is an effective material for elimination of bacteria [22]. Koch [23], believed that the amount of released formaldehyde and the antimicrobial activity of the sealer was affected by the mixing ratios of sealer constituents, the time elapsed after mixing and the area-to-weight of the sealer. 

MTA Fillapex (Angelus, Londrina, PR, Brazil) is a new sealer that has been marketed recently [24]. The philosophy of manufacturing this sealer is the presence of mineral trioxide aggregate (MTA) in its chemical structure [25]. One of the properties of MTA that is claimed to be present in the MTA Fillapex sealer, is the alkaline pH and subsequent antibacterial activity [26]. 

Previous studies were carried out on the antibacterial properties of AH-26 [1-3] and MTA Fillapex [1, 4] had mainly used agar diffusion test with *E. faecalis*. According to Anumula *et al. *[27], contact test can be conducted with two different methods; direct method to measure the antibacterial properties of the material and its diffused components, and the indirect method which measures the antibacterial effect of bacterial incubation period only.

Considering the differences in the materials and techniques used in studies mentioned above and discrepancies in their results and a lack of comprehensive studies on the wide range of microorganisms, the aim of the present study was to compare the antimicrobial effects of these two sealers on *Lactobacillus acidophilus *(*L. acidophilus*), *Lactobacillus casei *(*L. casei*), *Staphylococcus aureus* (*S. aureus*) and *E. faecalis* using the direct and indirect contact test at 24-,48- and 72-h and 7-day intervals after mixing.

## Materials and Methods

In the present study, the antibacterial properties of two commonly used sealers were evaluated: MTA Fillapex (Angelus, Londrina, PR, Brazil; Lot No: 15824) and AH 26 resin-based sealer (Dentsply, Tulsa Dental, Tulsa, OK, USA; Lot Number 0305001193).

The following bacterial species that are considered the etiologic agents for dental infections were evaluated and used for the evaluation of antibacterial properties of test sealers: *L. acidophilus* (ATCC4356), *L. casei* (ATTCC 39392), *S. aureus* (ATCC 25923) and *E. faecalis* (ATCC 29212).

All the microorganisms were standard microbial strains, selected from the stocks stored at -70^°^C and cultured again in blood agar culture medium, with their fresh cultures being used for the purpose of the study. All strains were provided from Iranian Research Organization for science and technology (IROST). All the microbial samples were cultured again in tryptic soy broth (TBS) medium (Difco Laboratories, Detroit, Mich., USA) for 48 h before evaluation. The results of the tests were recorded in terms of turbidity, which was determined visually using a spectrophotometer. 

***Contact test***

The present methodology was obtained from the study by Slutzky-Goldberg *et al. *[28]. The direct contact test was carried out in terms of turbidity of bacterial suspensions in a 96-well micro-titer plates and the results were determined at a wavelength of 600 nm in a spectrophotometer (BioTech Instruments, Inc, Winooski, VT, USA) after being cultured at 37^°^C [28]. 

Three 96-well micro-titer plates with smooth bases were selected and classified in groups so that the antimicrobial effects of sealers could be evaluated at different time intervals. 

In the direct technique the side wall of the wells were coated with 25 mL of the sealer to be tested and care was taken not to carry any amount of the material to the bottom of the well because it prevents necessary measurements for determination of turbidity in post-culturing stages by causing false positive results. Microbial suspensions were prepared separately, consisting of a solution of physiologic serum with the microbe-containing culture medium. Then 10 µL of the microbial suspension at a concentration of approximately 10^6^ were placed on the surface of the sealers in the wells. Then each plate was sealed and held vertically to evaporate the solution containing the bacterial species. To this end, the plates were incubated in 37^°^C for 1 h to ensure the direct contact of microbes with the sealer. Then 245 µL of the TSB solution was added and shaken gently for 2 min. 

In the indirect technique, 15 µL of the solution was retrieved and transferred to another well that contained 215 µL of the fresh culture medium. Therefore, it was possible to evaluate bacterial growth in direct contact with sealer (direct method) and without sealer (indirect method) [27].

The plates were incubated at 37^°^C for 24 h and readings were carried out at 600 nm so that changes in bacterial growth could be detected. The experiments were repeated for three times for each well in order to ensure accuracy of results. After mixing these tests were repeated at 24-, 48- and 72-h intervals and after 7 days [29]. 

***Data analysis***

Data were analyzed with descriptive statistical methods (mean±SD) and repeated-measures ANOVA followed by post hoc Bonferroni test, using SPSS software (SPSS version 20, SPSS, Chicago, IL, USA). Statistical significance was set at 0.05.

## Results

Tables 1 and 2 present the mean±SD of spectrophotometer readings regarding the effects of AH 26 and MTA Fillapex sealers on different bacterial species with the use of direct and indirect techniques. Tables 3 and 4 show the two-by-two comparison of different bacteria and different time intervals.

The antimicrobial effects of both sealers on the bacterial species in question decreased over time. The antimicrobial effects of MTA Fillapex on *E. faecalis* and AH 26 sealer on *L. acidophilus* did not exhibit significant differences over time. MTA Fillapex exhibited the lowest antibacterial effect on *E. faecalis*, with a similar effect on *S. aureus*, *L. acidophilus* and *L. casei*. AH 26 exhibited the lowest antibacterial effect on *E. faecalis* and *S. aureus*, with the highest antibacterial effect on *L. acidophilus* and *L. casei.* In addition, MTA Fillapex exhibited a significantly lower antibacterial effect on all the 4 bacterial species compared to AH 26 sealer. 

The antimicrobial effects of both sealers on the bacterial species in question decreased over time. Only, the antimicrobial effect of MTA Fillapex sealer on *L. acidophilus* and *L. casei* did not exhibit significant differences over time. MTA Fillapex exhibited the highest antibacterial activity on *E. faecalis*, with the least effect on *L. acidophilus*. AH 26 exhibited the highest antibacterial effect on* E. faecalis* and *S. aureus*, with the least effect on *L. acidophilus* and *L. casei.* In addition, both sealers had a similar antibacterial effect on all the four bacterial species.

## Discussion

It is not possible to completely eliminate microorganisms from the root canal system, even with debridement, shaping and irrigation of the root canals with antimicrobial agents. Therefore, use of root filling materials with antimicrobial activity might help achieving this goal [1, 2]. 

The microorganisms tested in the present study were either true endodontic pathogens or associated with persistent endodontic diseases [30]. Despite the fact that aerobic and facultative microorganisms usually constitute a minor proportion of primary endodontic infections, they are found with higher frequencies in cases with protracted treatment, in flare-ups and in endodontic failures [31, 32]. 

Therefore, *E. faecalis* was used in the present study. Also *S. aureus *was used in the present study because it is a standard organism in antimicrobial tests [33].

In direct contact test, there is a direct contact between the microorganism and the test material. This method was almost independent of diffusion and solubility properties of both materials and the test media [34]. Contrary to the indirect test, the direct contact test can show the antibacterial activity of insoluble components [27]. 

**Table 1:** Mean (SD) of spectrophotometer readings regarding the antibacterial effects of AH 26 and MTA Fillapex sealers at different times

	**Bacteria**	**Sealer**	**24 h**	**48 h**	**72 h**	**7 days**	***P-*** value*
**Direct**	***Staphylococcus aureus***	**AH 26**	1.611 (0.14)	1.336 (0.23)	1.082 (0.24)	0.852 (0.143)	0.007
		**MTA Fillapex**	1.717 (0.08)	1.550 (0.05)	1.364 (0.15)	1.245 (0.19)	0.009
		***P-*** value**	0.24	0.045	0.04	0.02	
	***Enterococcus faecalis***	**AH 26**	1.810 (0.04)	1.353 (0.099)	0.821 (0.297)	0.734 (0.285)	0.001
		**MTA Fillapex**	2.72 (0.072)	2.653 (0.061)	2.363 (0.340)	2.13 (0.472)	0.075
		***P-*** value**	0.000	0.000	0.000	0.000	
	***Lactobacillus acidophilus***	**AH 26**	0.961 (0.14)	0.854 (0.086)	0.639 (0.188)	0.663 (0.137)	0.066
		**MTA Fillapex**	1.62 (0.206)	1.40 (0.053)	1.42 (0.120)	1.30 (0.10)	0.012
		***P-*** value**	0.000	0.005	0.003	0.000	
	***Lactobacillus casei***	**AH 26**	1.324 (0.22)	1.03 (0.098)	0.585 (0.036)	0.714 (0.065)	0.000
		**MTA Fillapex**	1.80 (0.204)	1.79 (0.285)	1.68 (0.339)	1.328 (0.184)	0.001
		***P-*** value**	0.020	0.004	0.000	0.000	
**Indirect**	***Staphylococcus aureus***	**AH 26**	2.392 (0.08)	2.233 (0.057)	1.90 (0.104)	1.97 (0.086)	0.000
		**MTA Fillapex**	2.291 (0.05)	2.195 (0.081)	1.998 (0.104)	1.927 (0.09)	0.009
		***P-*** value**	0.14	0.66	0.30	0.62	
	***Enterococcus faecalis***	**AH 26**	2.67 (0.208)	2.142 (0.124)	2.07 (0.151)	1.86 (0.057)	0.001
		**MTA Fillapex**	2.334 (0.08)	2.206 (0.004)	2.142 (0.01)	1.973 (0.046)	0.000
		***P-*** value**	0.05	0.42	0.49	0.07	
	***Lactobacillus acidophilus***	**AH 26**	2.23 (0.152)	2.08 (0.167)	1.85 (0.036)	1.64 (0.241)	0.001
		**MTA Fillapex**	1.967 (0.153)	1.867 (0.252)	1.800 (0.361)	1.733 (0.34)	0.785
		***P-*** value**	0.10	0.26	0.83	0.69	
	***Lactobacillus casei***	**AH 26**	2.03 (0.120)	1.85 (0.100)	1.84 (0.03)	1.78 (0.09)	0.040
		**MTA Fillapex**	2.067 (0.306)	1.922 (0.357)	1.879 (0.002)	1.797 (0.016)	0.338
		***P-*** value**	0.76	0.71	0.43	0.54	

In the present study, evaluation of the antimicrobial properties of MTA Fillapex sealer showed that in the direct technique time affected the antimicrobial activity of the sealer except for its effect on *E. faecalis. *In the indirect technique, too, the antimicrobial activity of MTA Fillapex sealer was affected by time except for its effect on* L. acidophilus* and *L. casei*. The antimicrobial effect of sealers in the direct technique was higher than the indirect technique, which might be attributed to the fact that in the indirect technique, the sealer need a longer time to exert its effect on microorganisms, because indirect technique demonstrates incubation period of antibacterial materials [27]. Ustun *et al.* [35] showed that MTA Fillapex sealer did not exhibit any antibacterial effect up to 24 h. However, at 7- and 30-day intervals it preserved its antibacterial activity against *E. faecalis*, consistent with the results of the present study, despite differences in materials and study procedures. 

Faria-Junior *et al.* [26] showed that MTA-Fillapex sealer preserved its antimicrobial effect on bacterial biofilm, which was higher than the effect of AH Plus. Preservation of the antimicrobial activity at 2- and 7-day intervals in the study above is consistent with the results of the present study. 

A study by Morgental *et al.* [36] showed that MTA Fillapex had antibacterial effect on *E. faecalis* before setting but after setting, despite the high pH it did not possess this property. The results of the present study did not coincide with the study above. Despite the fact that the results of these two studies cannot be directly compared, the reason for such discrepancy between the results might be the differences between the two laboratory techniques. 

The results of the present study showed that AH 26 in the direct technique was effective against all the microorganisms over time except against *L. acidophilus* in the indirect technique, and time passing increased the antibacterial properties of the sealer. 

Mohammadi *et al.* [29] reported that AH 26 sealer preserved its antibacterial activity from 24 h up to 7 days, which is consistent with the results of the present study. The results of the studies by Al-Khatip [37] and Willershausen *et al. *[38] showed that AH 26 sealer has significant antibacterial activity for at least 35 days. The results of the present study were consistent with those of the studies above. 

**Table 2:** Mean (SD) of spectrophotometer readings regarding the antibacterial effects of test sealers using direct and indirect

		**MTA Fillapex**	**AH 26**	***P*** -value**
**Direct**	***Staphylococcus aureus***	1.468 (0.218)	1.220 (0.339)	0.045
	***Enterococcus faecalis***	2.465 (0.318)	1.180 (0.489)	0.000
	***Lactobacillus acidophilus***	1.437 (0.144)	0.778 (0.184)	0.000
	***Lactobacillus casei***	1.649 (0.217)	0.911^a^ (0.318)	0.000
	***P*** -value*	0.000	0.008	
**Indirect**	***Staphylococcus aureus***	2.102 (0.177)	2.123^a^ (0.216)	0.799
	***Enterococcus faecalis***	2.164 (0.137)	2.187^a^ (0.331)	0.824
	***Lactobacillus acidophilus***	1.8410 (0.261)	1.956^ab^ (0.252)	0.285
	***Lactobacillus casei***	1.909 (0.219)	1.873^b^ (0.104)	0.615
	***P*** -value*	0.001	0.009	

**Table 3:** Pairwise comparison of groups

		**Time 1**	**Time 2**	***Staphylococcus aureus***	***Enterococcus faecalis***	***Lactobacillus acidophilus***	***Lactobacillus casei***
**MTA Fillapex**	**Direct**	**24 h**	**48 h**	0.04		<.000	0.79
		**48 h**	**72 h**	0.03		0.43	0.09
		**72 h**	**7 days**	0.23		0.07	0.03
	**Indirect**	**24 h**	**48 h**	0.15	<.000	0.42	
		**48 h**	**72 h**	0.02	<.000	0.42	
		**72 h**	**7 days**	0.5	0.02	0.06	
**AH 26**	**Direct**	**24 h**	**48 h**	0.03	0.01		0.22
		**48 h**	**72 h**	0.02	0.03		0.01
		**72 h**	**7 days**	0.06	0.66		0.15
	**Indirect**	**24 h**	**48 h**	0.18	0.03	0.08	0.02
		**48 h**	**72 h**	0.01	0.05	0.16	0.94
		**72 h**	**7 days**	0.18	0.06	<.000	0.24

**Table 4:** Pairwise comparison of bacterial species

		**MTA Fillapex**		**AH 26**	
		**Direct**	**Indirect**	**Direct**	**Indirect**
***Staphylococcus aureus***	***Enterococcus faecalis***	<0.000	1	1	
***Staphylococcus aureus***	***Lactobacillus acidophilus***	1	0.019	021	
***Staphylococcus aureus***	***Lactobacillus casei***	0.385	0.152	0.219	
***Enterococcus faecalis***	***Lactobacillus acidophilus***	<0.000	0.002	0.044	
***Enterococcus faecalis***	***Lactobacillus casei***	<0.000	0.023	0.403	
***Lactobacillus acidophilus***	***Lactobacillus casei***	0.185	1	1	

Comparison of the antibacterial effects of the two sealers showed that the antimicrobial effect of MTA Fillapex in the direct technique on 4 evaluated microorganisms at 24-, 48- and 72-h and 7-day intervals was less than that of AH-26 sealer except for its effect on *S. aureus* at 24-h interval, with the similar effect of both sealers. In the indirect technique, the antimicrobial effect of both sealers was similar at all the intervals of the study and on all the microorganisms. 

Ehsani *et al.* [39] used agar diffusion test and showed that the antibacterial effect of AH 26 sealer on *E. faecalis* and *Lactobacillus* was significantly higher than that of MTA Fillapex sealer, which is consistent with the results of the present study. Madani *et al.* [40] used the agar diffusion test and reported that the antibacterial activity of MTA Fillapex sealer on *E. faecalis *was longer than that of AH 26 after a 24-h time interval, contrary to the results of the present study. Such discrepancy might be explained by differences in materials and methods or genetic differences in *E. faecalis* species evaluated in the present study. The clinical relevance of the different findings of direct and indirect method might be the inability of the sealer to fulfill all anatomical sites of root canal system. Therefore the findings of indirect method may demonstrate anatomical sites unreachable to sealer.

## Conclusion

The results highlighted the importance of complete filling of the root canal system and suggest AH 26 sealer according to its higher antibacterial properties.
